# Infection and treatment immunizations for successful parasite vaccines

**DOI:** 10.1016/j.pt.2013.01.003

**Published:** 2013-03

**Authors:** Francisca Mutapi, Peter F. Billingsley, W. Evan Secor

**Affiliations:** 1 Institute for Immunology and Infection Research, Centre for Immunity, Infection and Evolution, School of Biological Sciences, University of Edinburgh, Edinburgh, EH9 3JT, UK; 2 Sanaria Inc., Rockville, MD 20850, USA; 3 Division of Parasitic Diseases and Malaria, Centers for Disease Control and Prevention, Atlanta, GA 30329, USA

**Keywords:** immunization, treatment, vaccine, malaria, schistosomiasis, parasite

## Abstract

Since the advent of techniques for the expression of recombinant peptide antigens, the availability of human vaccines for parasitic diseases has been ‘imminent’. Yet vaccines based on recombinant proteins are still largely aspirations, not realities. It is now apparent that vaccine development needs additional knowledge about host protective immune response(s), antigen characteristics, and the delivery required to induce those responses. The most successful immune protection against parasites has been generated by infection and treatment, the induction of protective immunity by truncating the course of an infection with drug treatment. Here, we consider the characteristics of an effective, protective anti-parasite vaccine and propose a conceptual framework to aid parasite vaccine development using malaria and schistosomiasis as examples.

## Development of protective immune responses resulting from infections

Exposure to pathogens allows vertebrate hosts to mount pathogen-specific acquired immune responses that sometimes protect against subsequent infection, forming the basis of vaccinology [1]. The original observation that protection often succeeds infection and recovery led to the artificial induction of immunity by infection with attenuated parasites 2, 3, which triggered tremendous interest in the nature and development of naturally acquired protective immunity and characterization of measureable markers of immune protection. The broad range of veterinary [3] and human [4] vaccines against bacterial and viral pathogens are predominantly live attenuated or inactivated pathogen formulations (Table 1). Similarly, a significant proportion of protozoan vaccines against economically significant veterinary parasites (e.g., *Theileria*) of livestock and companion animals are based on inoculation with attenuated or drug treated parasites. In humans, the most widely used ‘vaccination’ for a parasitic infection is the practice of leishmanization [5], where children are inoculated with parasite-containing exudate from a cutaneous *Leishmania* sore in a location typically covered by clothing. The resulting, self-limiting lesion provides protection against subsequent infections that might otherwise form a disfiguring ulceration on an exposed area. However, no vaccines against parasitic infections are licensed for human use. This is at least in part attributable to the antigenic complexity of parasites, arising from multiple life cycle stages, immune evasion strategies, and use of intermediate and reservoir hosts. Unfortunately, obtaining adequate numbers of parasites, attenuated or otherwise, of consistent and acceptable quality to use in vaccinations is highly challenging, as demonstrated by recent studies of the *Plasmodium falciparum* attenuated sporozoite vaccine (PfSPZ vaccine) in humans [6]. Nevertheless, the recent Phase 1 trial demonstrating that injection of cryopreserved *P. falciparum* sporozoites can be used in controlled human malaria infections will greatly facilitate this research in the future [7].

**Table 1:** Currently licensed human vaccines^a^

Vaccine	Common name/combination vaccine	Pathogen	Type of vaccine
Anthrax		Bacteria	Subunit^b^
Chicken pox	Varicella	Virus	Live, attenuated^c^
Cholera			Inactivated^d^
Diptheria	DPT	Bacteria	Inactivated toxin
Haemophilus influenza type B	Hib	Virus	Conjugate^e^
Hepatitis A		Virus	Inactivated
Hepatitis B		Virus	Subunit
Human papillomavirus	HPV	Virus	Subunit
Influenza vaccine		Virus	Live, attenuated
Japanese encephalitis vaccine			Inactivated
Measles	MMR	Virus	Live, attenuated
Mumps	MMR	Virus	Live, attenuated
Rubella	MMR	Virus	Live, attenuated
Pertusis	Whooping cough (DPT)		Subunit
Pneumococcal infections	Meningitis and pneumonia Meningococcus	Bacteria	Subunit
Polio		Virus	Inactivated
Rabies		Virus	Inactivated
Rotavirus		Virus	Live, attenuated
Small pox		Virus	Attenuated (Sabin polio vaccine) Inactivated (Salk polio vaccine)
Shingles	Herpes zooster	Virus	Live, attenuated
Tetanus	DPT	Bacterial toxin	Inactivated toxin
Tuberculosis	Bacilli Calmette–Géurin (BCG)	bacteria	Live, attenuated
Typhoid		bacteria	Inactivated
Yellow fever		virus	Live, attenuated

a Table adapted from [5] and definitions adapted from http://www.niaid.nih.gov/topics/vaccines/understanding/pages/typesvaccines.aspx.

b Subunit vaccine: a vaccine made up of only the antigens that best stimulate the immune system. They are made in one of two ways: either by chemical extraction of the native antigen, the whole organism, or as recombinant proteins expressed in other organisms (e.g., bacteria), in which case they would be termed ‘recombinant subunit vaccines’.

c Live attenuated vaccine: a vaccine made from the living microbe that has been weakened in the laboratory so it cannot cause disease but may still be able to replicate in the host.

d Inactivated vaccine: a vaccine made by killing the disease-causing microbe with chemicals, heat, or radiation.

e Conjugate vaccine: a vaccine created by covalently attaching a poorly immunogenic antigen (e.g., a polysaccharide) to a carrier protein thereby conferring the immunological attributes of the carrier to the attached antigen. This type of vaccine is a special type of subunit vaccine.

**Figure 1:**
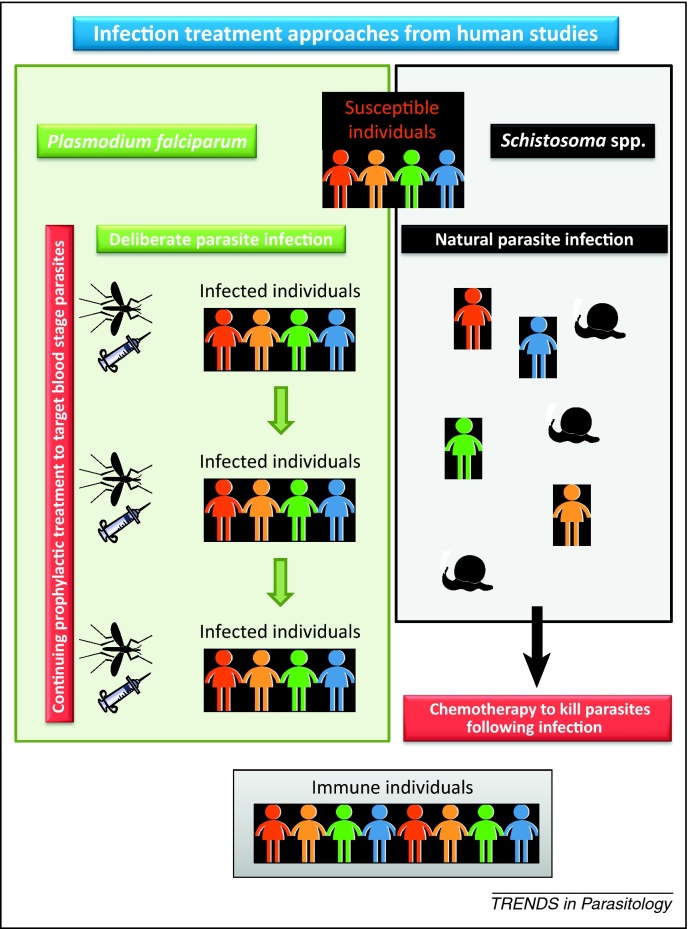
Approaches to infection and treatment vaccinations for malaria and schistosomiasis. Vaccination by infection and treatment (I&T) against malaria is achieved by providing chemoprophylaxis to susceptible individuals followed by spaced treatments with sporozoites delivered by mosquito bite or needle and syringe. Under these conditions, parasites develop in the liver but do not go beyond early blood stage infections. After final treatment, the chemoprophylaxis is withdrawn and individuals demonstrate increased immunity against future challenges. Vaccination by I&T against schistosomiasis is based upon infections acquired naturally followed by treatment with praziquantel or natural worm death, resulting in individuals with decreased susceptibility to future exposure.

An alternative to infection with attenuated parasites is the infection and treatment (I&T) approach where immunity is induced by the release of antigens from parasitic infections that are treated or naturally die in the host (Figure 1). One of the most striking examples of the effect of previous infection on subsequent protection is the relative resistance to symptomatic malaria in older children and adults who have grown up in areas endemic for *P. falciparum*. Recently, an I&T trial for malaria was performed by exposing volunteers who were receiving chloroquine prophylaxis to *P. falciparum* sporozoites. The chemoprophylaxis with sporozoites (CPS) protocol succeeded in inducing sterile immunity in all immunized participants and was maintained in four of six participants for >2 years [8]. An I&T effect is also observed in schistosome infections as praziquantel treatment of persons infected with *Schistosoma haematobium* or *Schistosoma mansoni* can induce partially protective immunity against subsequent infections 9, 10.

Another outcome of I&T is that individuals from areas where they are likely to have been exposed to malaria or schistosome antigens early in life tend to have a lower risk of developing severe pathological consequences such as cerebral malaria or hepatosplenic schistosomiasis, respectively. Protective mechanisms against pathology are poorly understood but are hypothesized to involve induction of different regulatory or memory immune responses. In addition to modulation of pathology in subsequent infections, I&T effects on host immune responses are also instructive with respect to development of defined antigen vaccines. Most vaccine recipients in endemic areas are likely to have had some exposure to the parasite, leading to reactions during immunization that may differ from those of parasite naïve vaccine trial participants. For example, the Phase I clinical trial evaluating the vaccine against human hookworm using *Ancylostoma* secreted protein (ASP-2) was discontinued when vaccination induced urticarial reactions in people with pre-existing IgE responses to ASP-2 [9]. No such adverse events have been reported in I&T.

Similar to inoculation with attenuated parasites, I&T has limitations that may preclude it from being a feasible public health tool; for some parasite species, it may not be possible to generate sufficient quantities of infectious stage parasites to vaccinate the millions of people exposed to these infections. Nevertheless, I&T approaches provide key answers to some fundamental intellectual and practical questions for successful vaccine development. By concentrating on the principles of classical vaccination, we describe how I&T protocols have overcome some of the challenges of using recombinant protein immunizations.

## Desirable I&T characteristics for successful vaccines

Parasites causing the greatest morbidity and disease typically induce a more or less protective immunity very slowly. Reasons for this include poor immunogenicity of individual antigens, poor protective immunity of major antigens, antigenic variation (protozoa), antigen polymorphism, immune evasion, immunomodulation of effector responses, and/or the requirement for a threshold amount of antigen which is released more easily upon treatment than from natural parasite death 10, 11, 12, 13, 14. I&T approaches have overcome some of these parasite survival strategies. Several important characteristics that underlie their success are discussed below.

### The pathogen must be immunogenic

Parasites successfully controlled by I&T are immunogenic during natural infections. *Echinococcus granulosus* onchospheres provoke a high degree of protective immunity, which is the basis of a highly effective vaccine in lambs (90% protection [15]) and offering great potential as a human parasite vaccine [16]. By contrast, vaccine development against *Fasciola hepatica* and *Fasciola gigantica* is hampered by their inability to induce immunity in their natural hosts, even after repeated infections, suggesting low immunogenicity of these flukes [2]. Parasites might be immunogenic but still infect the host if the host is unable to recognize the pathogen or mount a protective immune response during the parasite's immune-susceptible period. For example, infective stages of schistosomes, filarids, and hookworms are susceptible to immune attack but migrate and mature before effective immune responses develop. Subsequent infections may be prevented but only after the initial parasites become established [17]. Furthermore, adult schistosomes avoid the host's protective immunity through evasive mechanisms such as rapid membrane turnover, host mimicry, and masking themselves with host proteins [18].

In the CPS and PfSPZ–CVac studies, the timing of drug treatment allows full development of the liver stage parasites, thereby increasing the number and diversity of parasite antigens. It also eliminates the parasites before the onset of disease [19] and prevents inhibition of anti-liver stage cellular immunity that would otherwise occur during blood stage infections [20]. Additionally, with the increased complexity of whole parasite antigens against parasite stages for which protective immunity might not otherwise develop, I&T offers great potential for strain-transcending protection.

However, protozoan parasites can vary the antigens seen by host immune systems through mechanisms including transcriptional and epigenetic control (*in situ* switching, e.g., *P. falciparum* or *Giardia lamblia*) or through gene conversion (unidirectional recombination, e.g., *Trypanosoma brucei* and *Babesia bovis*) [21]. I&T may overcome antigenic variation and immune avoidance by inducing immunity to many antigens of several parasite strains/variants. Such broad coverage is very challenging to achieve with a recombinant vaccine, even if it is multivalent, such as the AMA1 [22] or MSP-1 [23] vaccine candidates. To date, antigenic switching has not been demonstrated in helminths, although a micro-exon mechanism for potentially generating antigenic variation is present in the schistosome genome [24].

### Inducing protective effector, rather than regulatory responses

Individuals infected naturally with schistosomes or malaria parasites eventually develop effector responses that may confer protection despite also stimulating regulatory responses. Schistosome infection intensities are associated with the balance between protective and regulatory responses, which is affected by host age [25]. An often overlooked aspect of protective immunity is how effector responses surpass regulatory responses during natural infection and progress to long-lived memory B and T cells. Understanding this phenomenon may help unlock the door to successful vaccine design.

Not all immune responses result in parasite killing or resistance to re-infection. Indeed, some parasite evasive mechanisms may divert the immune system to respond against ‘decoy’ antigens. In *Plasmodium*, T cell mimotopes are protein variants of parasite antigens (altered peptide ligands) that prevent development of memory T cell effector functions of cytotoxic lymphocytes [26]. Similarly, carbohydrate epitopes on schistosome cercariae and egg antigens predominantly induce IgM and IgG2 antibodies, which are not as efficacious against schistosomulae as other antibody subclasses 27, 28 and skew the immune system towards anergy [29]. Why the host maintains these ineffective responses is unknown but there may be homeostatic reasons for maintaining them [30]. Thus, the value of these responses must be considered before disregarding them entirely. Alternatively, certain responses may be regulatory and suppress protective immunity, such as the AgEm2 carbohydrate in *E. granulosus* that interferes with antigen presentation and cell activation [31].

Amelioration of autoimmunity in rodents by *Plasmodium* suggests that blood stage parasites induce immunosuppression. This was confirmed in humans as blood stages of *P. falciparum* suppress T lymphocyte reactivity to malarial and unrelated antigens [32]. The several immunosuppressive strategies employed by *Plasmodium* parasites include utilization of T cell mimotopes to inhibit T cell activation, induce anergy, or shift the T cell phenotype [33]; alteration of antigen presentation by impairing dendritic cell function and maturation [34]; and induction of regulatory T cells [35]. By contrast, the *P. falciparum* I&T trial in humans resulted in equivalent or better protection than that produced by irradiated sporozoite immunizations [36]. I&T exposes the host to all pre-erythrocytic stages, allowing effector responses to be mounted against a broader range of antigens, while limiting exposure to the pathogenic and immunosuppressive asexual blood stages 37, 38. In natural infections, people are typically infected with *Plasmodium* from a single mosquito bite and treated based on clinical symptoms or diagnosis, at which time the blood stage parasites may be suppressing pre-erythrocytic immunity. In successful *Plasmodium* CPS, the drug was administered prophylactically, including during inoculation of sporozoites from 15 mosquitoes [39]. This requirement to curtail exposure to blood stage parasites for full protection is supported by development of protective acquired immunity in children receiving treatment that restricts the development of symptomatic malaria [40]. Thus, successful vaccination must overcome the effects of regulatory responses that are stimulated by certain parasite life cycle stages. I&T for *Plasmodium* has achieved this by minimizing immune exposure to immunosuppressive blood stage parasites. How or even if this is achieved in people who develop natural resistance to malaria is unclear.

Experimental and natural helminth infections are associated with immunoregulatory responses that polarize CD4^+^ T cells towards a T helper 2 (Th2) phenotype (production of interleukins 4, 5, and 13, secretion of IgE and IgG4 by plasma cells, and activation of eosinophils and mast cells), and immunosuppress worm-specific [41] and general [42] immune responses. Helminth parasites modulate both innate and adaptive arms of the immune system, targeting both humoral and cellular responses [43]. Regulatory responses are characterized by suppressive cytokines [interleukin (IL)-10 and transforming growth factor β (TGF-β)] produced by natural and adaptive regulatory T (Treg) cells 44, 45 that can block resistance to schistosome reinfection in an animal model [46]. Experimental studies clearly show that whilst Treg cells play an important role in shaping anti-schistosome responses, the ‘regulatory’ arm of the immune axis extends beyond this population [47] including other cells such as Th1 cells and macrophages 48, 49 (e.g., alternatively activated macrophages [50]). The influence of these regulatory responses on the development of resistance in human hosts is still under investigation. Cross-inhibition between effector CD4^+^ T cell subsets (Th1, Th2, and Th17) also means that effector cytokines [interferon (IFN)-γ, IL-4, and IL-21) are required to maintain a balanced acquired immune response 51, 52, which is associated with protective immunity against infection/re-infection 53, 54. When schistosome infection is cleared by drug treatment, immune reactivity can increase shortly afterwards, presumably in response to: (i) antigen release from dead parasites [14], (ii) reversal of hyporesponsiveness [55], or (iii) an increased effector T (Teff):Treg ratio [56]. Thus, success of I&T in human schistosomiasis may result partly from the treatment-induced increase in effector responses relative to regulatory responses.

### The effective dose: protective, non-pathological immune responses

Quantitative studies in human schistosomiasis show that immunosuppression alone does not explain the effects of age on infection intensity observed in schistosome endemic areas [57]. Rather, protective immunity develops only after exposure surmounts a threshold of parasite immunogens 14, 58. During natural schistosome infection, where adult worms survive for 3–7 years [59], it may take several years for the threshold to be reached as worm death events are spread out over time, only occasionally exposing the host to adult antigens that induce cross-reactive protection against invading schistosomulae. Similarly, it may take several rounds of *Plasmodium* infection for sufficient amounts of antigens from different circulating strains of the parasite to stimulate development of partially protective immunity [60]. In areas with higher transmission rates, *Plasmodium* and schistosome infection prevalence rates peak and decline at an earlier age. This pattern, first described in *Plasmodium* in 1949 [61], and later confirmed for other parasitic infections, is termed the ‘peak shift’ and has been attributed to development of acquired immunity [62]. Thus, protective immune responses develop earlier in high rather than low transmission areas [63], possibly due to immune stimulation after death of sufficiently large numbers and/or strains of parasites. The greatest exposure occurs when parasites are killed by treatment, providing in a single event the threshold exposure seen only over time or not at all in naturally resolving infections [64]. Praziquantel treatment for schistosomiasis qualitatively and quantitatively increases the antigens recognized by the host's immune system, mirroring the natural changes observed with host age 14, 64. The complexity of an immune response depends on the relative frequency of antigen-specific B and T cells, the levels of antigen present, and the period during which the antigen remains available to antigen-presenting cells. Thus, the antigen dose is critical at several stages in the generation of a response.

Immunization with irradiated *P. falciparum* sporozoites or irradiated cercariae suggest that high antigen doses are required to stimulate protective responses [65]. Furthermore, low-dose stimulation can induce antigen-specific FOXP3^+^ regulatory responses in humans and promote the development of tolerance [66]. The interaction between the antigen dose/duration of antigen stimulation and the immune system in humans exposed naturally to parasitic diseases is less well studied compared with responses induced in primary responses. An important factor in chronic parasitic infections such as schistosomiasis is the persistence of antigenic stimulation. In general, the immunological outcome of persistent stimulation by low antigen doses differs from intermittent stimulation with high antigen doses and determines whether the outcome is tolerance, pathology, or protection. Chronic immune activation in helminth infections results in impaired signal transduction and anergy [67], contributing to hyporesponsiveness [54]. I&T against schistosomiasis might overcome this hyporesponsiveness by releasing a higher immunizing antigen dose, thus avoiding low-dose anergy, while removing potentially immunosuppressive adult worms. Repeated treatment provides the additional necessary ‘doses’ for improved protection by stimulating development of high-affinity antibodies, such as anti-schistosome IgE [68].

### Long-lasting induced immunity

Almost all current vaccines work through induction of serum or mucosal antibodies that block infection or interfere with invasion and proliferation. Although antibody half-life is ∼30 days, effective vaccine-induced protection persists beyond the time when antibodies should have disappeared. Possible reasons for this include boosting from natural infection, antigen retention in peripheral lymph nodes by follicular dendritic cells, presence of sequestered memory B cells in bone marrow sanctuaries, or maintenance of antigen-specific memory B cells by nonspecific B cell activators or idiotypic networks. The importance of natural boosting has been demonstrated in animal I&T protocols for protozoa infections (e.g., *Theileria* [3]) and may explain why protective immunity against parasites can be lost when people migrate from endemic areas and are no longer exposed [69].

Both antibodies and CD4^+^ T cells form crucial components of naturally acquired protective immunity against blood stage malaria [70]. The longevity of these responses is a subject of much debate, with the majority of earlier immunoepidemiological studies showing them to be short-lived, especially in children 71, 72, 73, and characterized by a decline in antibody titers in the absence of parasitemia 71, 74, 75. However, even in low transmission areas, *P. falciparum* and *Plasmodium vivax* can induce long-term protection, despite the fact that effector inflammatory responses are short-lived 76, 77. Serum antibody titers in low malaria transmission areas varied in breadth and magnitude, confirming that several malaria infections are required to induce long-lived effector responses that even then are only partially protective. The implications of this for vaccine development, especially for people resident in areas of high malaria transmission, remain to be investigated, and the mechanisms and generation of long-lived responses in humans also require further investigation. Malaria I&T provides clinical and parasitological protection against malaria for at least 2.5 years [8], compared with the short-lived, partially protective immunity in natural infections. Furthermore, malaria I&T provided longer lived responses than the subunit based RTS,S/AS01 for which protection did not last beyond 12 months, particularly for severe malaria [78]. Mechanistic studies of the protective immune responses resulting from I&T will be very informative for designing long-lasting vaccines.

Understanding the longevity of antibody responses in schistosomiasis is confounded by the effects of repeated infections. Long-lasting responses follow curative treatment of schistosomiasis [79], which may be stimulated by prolonged excretion of antigens from live and dead eggs trapped in host tissues [80]. Similarly, European travelers infected with schistosomes at a single time point had schistosome-specific cytokine responses 8 years after treatment [81]. The possibility of low-grade infection in these participants or cross-reactivity might also explain these observations [82]. However, it is difficult to extrapolate these findings to endemic populations where chronic antigen stimulation may result in different B and T cell dynamics [83].

### Long-term induced immunological memory

The generation and persistence of immunological memory after an initial encounter with a pathogen provides the basis for subsequent protection. However, little is known about memory responses from repeated or chronic antigen exposure. How each round of infection affects the generation and maintenance of the memory T cell pool is poorly understood. Similar to the protective immunity in the *P. falciparum* I&T trial, effector memory T cells associated with protection against re-infection were maintained throughout the 2.5-year follow-up period 8, 19. By contrast, memory effector cell responses in natural infections decline after 12 months [75]. Thus, I&T might promote the longevity of effector memory responses.

Although activation and differentiation of CD8^+^ and CD4^+^ cells are broadly similar, vaccination strategies designed to induce and boost different T cell memory subsets have important distinctions. CD4^+^ memory T cells require a higher antigen threshold and prolonged stimulation for activation than CD8^+^ memory cells [84]. Also, persistence of T cell memory after vaccination with small viral fragments is much shorter than that achieved with attenuated whole virus [85].This may reflect differential temporal dynamics of memory responses and may explain the improved performance of whole parasite vaccines or I&T compared with recombinant vaccines. Furthermore, attenuated sporozoite immunization may stimulate different T cell memory generation pathways in novel sites (i.e., the liver) compared with natural infections, resulting in longer lasting vaccine-induced T cell memory [86].

Long-term antigen persistence and exposure in chronic helminth infections affects the development of immunological memory [86]. Recent work in human schistosomiasis showed that although there were no differences in CD8^+^ T cells in schistosome-infected versus uninfected people, CD4^+^ T cell proportions were significantly lower in individuals with schistosomiasis [87]. The reduced memory CD4^+^ cells during chronic infection may result from the Hayflick limit (the number of times a cell will divide [88]), which can significantly affect the generation of immunological memory under persistent antigenic stimulation [89]. In human schistosomiasis, although praziquantel treatment leads to a significant decline in CD4^+^ memory T cell proportions, there is a pronounced increase in CD4^+^ memory cell replication [87]. This suggests that the nature of immunological memory following I&T may differ from what occurs naturally.

## Concluding remarks

The biological hurdles to successful vaccine development are clear but I&T successfully addresses many of them. Schistosome vaccine development is hindered by factors which include: (i) lack of vaccine candidates providing reproducible protection in experimental models and humans; (ii) limitations in current understanding of the nature, development, and maintenance of protective immune responses, particularly in people already exposed to schistosomes; and (iii) how to induce protective immune responses better and faster than what occurs naturally while at the same time avoiding pathology or tolerance [9]. Similar hurdles face malaria vaccine development. It will be almost 40 years from initial studies to a commercially available formulation of RTS,S/AS01. During this time, the scientific challenges have involved antigen identification, identification of suitable vaccine vehicle and adjuvants, optimization of dosage and boosting schedules, and definition of immune correlates [90]. The highly successful I&T study with *P. falciparum* has provided answers to some of these questions and the tools to answer more. The exciting developments in I&T will hopefully provide the springboard for a better understanding of induced rather than acquired immunity against parasites and offer a new platform for development of truly effective anti-parasite vaccines.

## Disclaimer statement

The conclusions in this paper are those of the authors and do not necessarily represent the views of the Centers for Disease Control and Prevention.
